# Reactions of Organic Disulfides and Gold(I) Complexes

**DOI:** 10.1155/MBD.1999.247

**Published:** 1999

**Authors:** Melanie DiLorenzo, Shantheni Ganesh, Lily Tadayon, Jinhua Chen, Mitchell R. M. Bruce, Alice E. Bruce

**Affiliations:** Department of Chemistry University of Maine Orono Maine 04469-5706 USA

## Abstract

Gold-thiolate/disulfide exchange reactions of (p-SC_6_H_4_Cl)_2_ with Ph_3_PAu(SC_6_H_4_CH_3_), dppm(AuSC_6_H_4_CH_3_)_2_, and dppe(AuSC_6_H_4_CH_3_) _2_ were investigated. The rate of reactivity of the gold-thiolate complexes with (p-SC_6_H_4_Cl)_2_ is: dppm(AuSC_6_H_4_CH_3_)_2_>> dppe(AuSC_6_H_4_CH_3_)_2_>Ph_2_PAu (SC_6_H_4_CH_3_). This order correlates with conductivity measurements and two ionic mechanisms have been evaluated. ^1^H NMR experiments demonstrate that in the reaction of dppm(AuSC_6_H_4_CH_3_)_2_ with (p-SC_6_H_4_Cl)_2_, the mixed disulfide, ClC_6_H_4_SSC_6_H_4_CH_3_, forms first, followed by the formation of (p-SC_6_H_4_CH_3_)_2_. The rate law is first order in (pp-SC_6_H _4_Cl)_2_ and partial order in dppm(AuSC_6_H_4_CH_3_)_2_. Results from electrochemical and chemical reactivity studies suggest that free thiolate is not involved in the gold-thiolate/disulfide exchange reaction. A more likely source of ions is the dissociation of a proton from the methylene backbone of the dppm ligand which has been shown to exchange with D_2_O. The implications of this are discussed in terms of a possible mechanism for the gold-thiolate/disulfide exchange reaction.


### REACTIONS OF ORGANIC DISULFIDES AND GOLD(I) COMPLEXES

Melanie DiLorenzo, Shantheni Ganesh, Lily Tadayon, Jinhua Chen, Mitchell R. M. Bruce* and Alice E. Bruce*

Department of Chemistry, University of Maine, Orono, Maine 04469-5706, USA

### ABSTRACT

Gold-thiolate/disulfide exchange reactions of $(p\text{-}SC_6H_4Cl)_2$ with $Ph_3PAu(SC_6H_4CH_3)$, dppm$(AuSC_6H_4CH_3)_2$, and dppe$(AuSC_6H_4CH_3)_2$ were investigated. The rate of reactivity of the gold-thiolate complexes with $(p\text{-}SC_6H_4Cl)_2$ is: dppm$(AuSC_6H_4CH_3)_2$>> dppe$(AuSC_6H_4CH_3)_2$ > $Ph_3PAu(SC_6H_4CH_3)$. This order correlates with conductivity measurements and two ionic mechanisms have been evaluated. $^1H$ NMR experiments demonstrate that in the reaction of dppm$(AuSC_6H_4CH_3)_2$ with $(p\text{-}SC_6H_4Cl)_2$, the mixed disulfide, $ClC_6H_4SSC_6H_4CH_3$, forms first, followed by the formation of $(p\text{-}SC_6H_4CH_3)_2$. The rate law is first order in $(p\text{-}SC_6H_4Cl)_2$ and partial order in dppm$(AuSC_6H_4CH_3)_2$. Results from electrochemical and chemical reactivity studies suggest that free thiolate is not involved in the gold-thiolate/disulfide exchange reaction. A more likely source of ions is the dissociation of a proton from the methylene backbone of the dppm ligand which has been shown to exchange with $D_2O$. The implications of this are discussed in terms of a possible mechanism for the gold-thiolate/disulfide exchange reaction.

### INTRODUCTION

Gold in the +1 oxidation state is a soft metal and is therefore expected to have less affinity for the "hard" sulfur in disulfides than for the "soft" sulfur in thiolates.[1] However, there are several reports in the literature that demonstrate that gold(I) can react with simple organic disulfides as well as the disulfide bonds in proteins.[2,3] The reaction of gold(I) and disulfides is potentially significant for the biochemistry of gold(I) drugs because of the importance of disulfide bonds in stabilizing protein structure and moderating biochemical reactions. There is added significance for rheumatoid arthritis where oxidative stress is believed to result in an increase in disulfide bonds in proteins.[2b]

Several years ago, we began a program to study whether phosphine gold(I) thiolate complexes would react with organic disulfides.[4] This study was motivated by the observation that dppm$(AuSC_6H_4CH_3)_2$ undergoes the gold-thiolate/disulfide exchange reaction shown in eq. 1.[4,5] There are

$$LL(AuSC_6H_4CH_3)_2 + (p\text{-}SC_6H_4Cl)_2 \rightarrow LL(AuSC_6H_4Cl)_2 + (p\text{-}SC_6H_4CH_3)_2 \quad (LL = \text{dppm, dppe}) \tag{1}$$

$$2\ LAuSC_6H_4CH_3 + (p\text{-}SC_6H_4Cl)_2 \rightarrow 2\ LAuSC_6H_4Cl + (p\text{-}SC_6H_4CH_3)_2 \quad (L = PPh_3) \tag{2}$$

several features that make this reaction noteworthy. It occurs at room temperature in $N_2$-purged $CH_2Cl_2$ or $CHCl_3$ solution while thiol-disulfide exchange reactions generally require elevated temperatures (100 °C), the presence of $O_2$, or prior deprotonation of the thiol.[6] Another interesting feature is that the corresponding reactions of disulfides and the mononuclear complex, $Ph_3PAu(SC_6H_4CH_3)$, or the binuclear complex, dppe$(AuSC_6H_4CH_3)_2$, with a longer bisphosphine backbone, do not readily occur under similar conditions.[4,5] These observations prompted us to undertake a more thorough investigation of the gold-thiolate/disulfide exchange reactions as shown in eqs. 1 and 2.

### MATERIALS AND METHODS

**Materials.** Solvents used in synthesis reactions were reagent grade from Baker or EM Science and used as received, except for dichloromethane which was dried and distilled from $P_2O_5$. Tetrahydrofuran was HPLC from Sigma-Aldrich. Deuterated solvents for NMR studies were purchased from Cambridge

Isotope Labs. Hydrogen tetrachloroaurate was received as a loan from Alfa Aesar (Johnson Matthey). Thiols ($p$-$HSC_6H_4CH_3$, $p$-$HSC_6H_4Cl$), phosphines ($PPh_3$, dppm, dppe), and $(CH_3O)_3PO$ were purchased from Aldrich and the disulfides, ($p$-$SC_6H_4CH_3)_2$ and ($p$-$SC_6H_4Cl)_2$ were purchased from Lancaster Synthesis. The disulfide, $ClC_6H_4SSC_6H_4CH_3$ was prepared according to published procedures.[7] The phosphine gold(I) thiolate complexes were prepared according to previously published methods.[8]

**Abbreviations.** The following abbreviations are used: $p$-tc = $p$-thiocresol, dppm = 1,2-bis(diphenyl)phosphinomethane, dppe = 1,2-bis(diphenyl)phosphinoethane, dmso = dimethysulfoxide, TBAH = tetra-N-butylammonium hexafluorophosphate.

**Instrumentation.** NMR spectra were recorded on a 300 MHz Varian Gemini XL-300 spectrometer. $^1H$ NMR spectra are referenced on the residual proton signal in the deuterated solvent. Conductance measurements were made by using a YSI Model 35 Conductance meter. The cell was a dip-type cell, #3403.

**Conductivity Experiments.** Conductance measurements were referenced against standard solutions of KCl in water.[9] The conductance of 15 mL of solvent, either methylene chloride or tetrahydrofuran, was measured in the cell prior to addition of the gold sample. Gold complexes were weighed out in 5-10 mg increments. After addition of each increment, the conductance was measured. Typically, a total of 40-60 mg of compound was used. All measurements were made in the air and at room temperature.

### RESULTS AND DISCUSSION

The gold-thiolate/disulfide exchange reactions, shown in eqs. 1-2, can be monitored by $^1H$ NMR.[5,10,11] In the reaction of dppm$(AuSC_6H_4CH_3)_2$ with disulfide (eq 1), the mixed disulfide, $ClC_6H_4SSC_6H_4CH_3$ begins forming immediately upon mixing. After 20 minutes, 35% of ($p$-$SC_6H_4Cl)_2$ is converted into $ClC_6H_4SSC_6H_4CH_3$. Although the reaction is initially fast, it gradually slows down so that after 1.5 hours, conversion of ($p$-$SC_6H_4Cl)_2$ to $ClC_6H_4SSC_6H_4CH_3$ is 50% complete and after 15 hours, it is 65% complete (see Table 1). The symmetrical disulfide, ($p$-$SC_6H_4CH_3)_2$, and the symmetrical gold product, dppm$(AuSC_6H_4Cl)_2$, also form later in the reaction. In contrast, reaction of $Ph_3PAu(SC_6H_4CH_3)$ or dppe$(AuSC_6H_4CH_3)_2$ with ($p$-$SC_6H_4Cl)_2$ under similar conditions produces no detectable amount of $ClC_6H_4SSC_6H_4CH_3$ [or ($p$-$SC_6H_4CH_3)_2$] during the first several hours. A small amount of reactivity is seen only over a long time period.

**Rate Studies.** Monitoring the $^1H$ NMR spectra at different initial concentrations of gold-thiolate and disulfide, allows for an estimation of the rate of reaction.[5,10,11] The rate law for the reaction of dppm$(AuSC_6H_4CH_3)_2$ with ($p$-$SC_6H_4Cl)_2$ is first order in disulfide but is only partial order in dppm$(AuSC_6H_4CH_3)_2$.[12] The partial order of the gold complex indicates the possibility of a pre-equilibrium whereby only a fraction of the gold complex present in solution is active towards the gold-thiolate/disulfide exchange reaction.

**Table 1.** Extent of Reaction of Disulfides with Gold Complexes and Molar Conductivity of Gold Complexes.

| Gold Complex[a] | Disulfide[a] | % Reacted in 15 hours | Molar Conductivity[b,c] |
|---|---|---|---|
| $Ph_3PAuSC_6H_4CH_3$ | ($p$-$SC_6H_4Cl)_2$ | 8 | 0.04 |
| dppe$(AuSC_6H_4CH_3)_2$ | ($p$-$SC_6H_4Cl)_2$ | 24 | 0.8 |
| dppm$(AuSC_6H_4CH_3)_2$ | ($p$-$SC_6H_4Cl)_2$ | 65 | 4 |

a. 4 mM in $CDCl_3$. b. Gold complex alone, 1 mM in $CH_2Cl_2$, c. Siemens $cm^2$ $mol^{-1}$.

**Conductivity.** To aid our interpretation of the rate data, we performed conductivity measurements on a series of complexes including $Ph_3PAu(SC_6H_4CH_3)$, dppe$(AuSC_6H_4CH_3)$, and dppm$(AuSC_6H_4CH_3)_2$.[5,10,11] As shown in Table 1, the conductivity of dppm$(AuSC_6H_4CH_3)_2$ in $CH_2Cl_2$ is an order of magnitude larger than the mononuclear complex and 5 times greater than the dppe complex. All complexes exhibit weak electrolyte behavior. In Figure 1, the conductivity of dppm$(AuSC_6H_4CH_3)_2$ is shown in comparison to [Au(*cis*-dppee)2][PF6], a 1:1 electrolyte. This comparison allows us to estimate that the percent ionization of dppm$(AuSC_6H_4CH_3)_2$ in $CH_2Cl_2$ is about 10%.[13] The correlation of the rate of the gold-thiolate/disulfide exchange reaction with conductivity led us to consider mechanisms involving ionic intermediates.

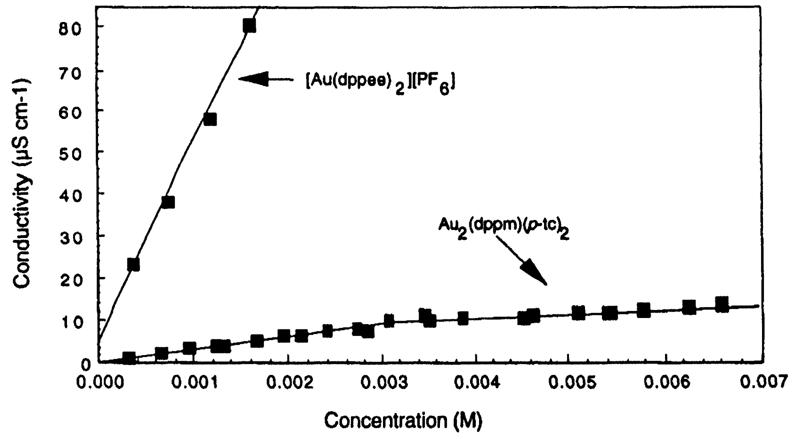

**Figure 1.** Conductivity (µ Siemens cm$^{-1}$) *vs.* concentration (Molar) in $CH_2Cl_2$ solutions of dppm$(AuSC_6H_4CH_3)_2$ and [Au(*cis*-dppee)2][PF6].

**Possible Ionic Intermediates.** Scheme 1 shows two possibilities for forming ionic intermediates: (a) dissociation of thiolate with formation of a cationic gold complex and thiolate anion or (b) dissociation of a proton from the dppm ligand with formation of an anionic gold complex and a proton.

**Scheme I** (R = *p*-$C_6H_4CH_3$)

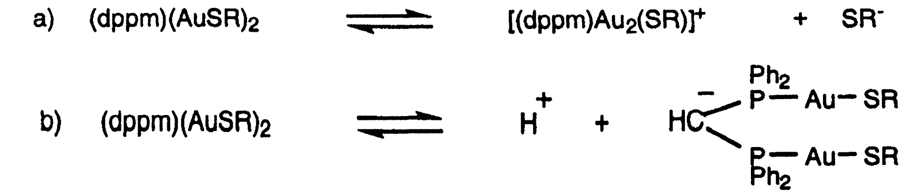

Pathway a in Scheme I would explain the data because thiolate/disulfide exchange reactions readily occur in solutions containing free thiolate.[6d-h] However, three lines of evidence suggest that this pathway is not important in the initial reactivity observed in the gold-thiolate/disulfide exchange reaction. The first line of evidence involves electrochemical tests for free thiolate. Cyclic voltammetry experiments on dppm$(AuSC_6H_4CH_3)_2$ show that there are two irreversible oxidation waves at ≈ +0.6 V and +1.6 V vs. SCE.[14] The first oxidation wave has been assigned as a sulfur-based oxidation on the thiolate ligand. In contrast, the free thiolate, *p*-thiocresolate, oxidizes irreversibly at ≈ 0 V vs. SCE.[15] Therefore if a thiolate ligand was dissociating from the gold complex (pathway a) it should be possible to detect it by oxidation. Figure 2 shows the results of two bulk electrolysis experiments conducted on solutions of dppm$(AuSC_6H_4CH_3)_2$ in 1.0 M TBAH/$CH_2Cl_2$. Figure 1a shows the total coulombs passed vs. time for a bulk electrolysis experiment done at +0.3 V vs. SCE using a Pt mesh working electrode. There is no sign of oxidation and the cyclic voltammogram of the solution is identical to that prior to electrolysis. For comparison, Figure 1b shows the bulk electrolysis conducted at +1.1 V vs. SCE (after the first oxidation wave for dppm$(AuSC_6H_4CH_3)_2$, but before the second) in which a significant amount of oxidation occurs.

The cyclic voltammogram of this solution shows that the first oxidation wave at +0.6 V has disappeared, but the peak at +1.6 V is still present.

Another test for free thiolate made use of the reaction between $(CH_3O)_3PO$ and thiolate, which is known to be rapid (eq. 3).[16] When $(CH_3O)_3PO$ is added to dppm$(AuSC_6H_4CH_3)_2$ in $d_6$-dmso (in an approximate 1:4 molar ratio), there is no reaction even after one week.[10]

$$(CH_3O)_3PO + {}^{-}SC_6H_4CH_3 \rightarrow (CH_3O)PO_2^{-} + CH_3SC_6H_4CH_3 \qquad (3)$$

Shaw and coworkers have studied ligand scrambling reactions of $R_3PAu(CN)$ to form $(R_3P)_2Au^+$ and $Au(CN)_2^-$. These reactions are unusual because they do not require the presence of excess ligand.[17] We tested our complexes for the presence of ligand scrambling to see if that might be contributing to the conductivity and reactivity with disulfide. Thus if a thiolate ligand was dissociating from the gold complex, then in a mixture of two different dinuclear gold complexes (which have very similar molar conductivities)[5] the thiolate ligands should exchange as shown in equation 4. This exchange would be detected by a shift

$$\text{dppm}(AuSC_6H_4CH_3)_2 + \text{dppm}(AuSC_6H_4Cl)_2 \rightleftharpoons 2\ \text{dppm}(AuSC_6H_4CH_3)(AuSC_6H_4Cl) \qquad (4)$$

in the *meta* hydrogens on the aromatic ring of the thiolate, relative to the pure samples. However, the $^1H$ NMR spectrum of a 1:1 mixture of dppm$(AuSC_6H_4CH_3)_2$ and dppm$(AuSC_6H_4Cl)_2$ in $CD_2Cl_2$ shows that the doublet peaks centered at 6.87 and 6.97 ppm, assigned to the *meta*-ring hydrogens in $SC_6H_4CH_3$ and $SC_6H_4Cl$, respectively, do not shift and are identical to the chemical shifts for each complex alone.[10,11]

An interesting feature in the $^1H$ NMR spectrum of dppm$(AuSC_6H_4CH_3)_2$ is that the methylene hydrogens on the dppm ligand appear as a broad triplet at ≈ 3.7 ppm (in $CD_2Cl_2$) and the chemical shift is concentration dependent.[8,10] This suggests that there is an equilibrium between two species involving the methylene hydrogens on the dppm ligand. To test this hypothesis, two equivalents of $D_2O$ were added to a solution of dppm$(AuSC_6H_4CH_3)_2$ in $CD_2Cl_2$. After 3 hours the methylene hydrogens on the dppm ligand have decreased by one-half and after 12 hours, the triplet signal has disappeared. Thus, pathway b in Scheme I appears to be a better explanation for the ionic intermediates present in solutions of dppm$(AuSC_6H_4CH_3)_2$.

There are several examples in the literature that illustrate the acidity of the methylene hydrogens in coordinated dppm.[18] However, deprotonation of a dppm ligand generally requires a strong base. An important feature of the dinuclear gold complex, dppm$(AuSC_6H_4CH_3)_2$, is that it exists in solution as a mixture of gold-gold bonded and nonbonded conformers.[4,8] The activation barrier for interconversion of these two conformers is about 10 kcal/mole, which agrees with other estimates of the gold-gold bond strength.[19] Our results suggest that the auriophilic Au-Au interaction in dppm$(AuSC_6H_4CH_3)_2$ perturbs the electronic structure enough to effect the acid-base properties of the methylene protons in dppm. Indeed there is precedent in the literature for this type of effect. Fackler, Schmidbaur, and coworkers recently reported that the basicity of a nitrogen atom in a TPA ligand bound to gold(I) is greater when there is a stronger Au-Au interaction. Thus the solid state Au-Au interaction is 0.2 Å shorter in (TPA)AuCl vs. (TPA-HCl)AuCl (where TPA is 1,3,5-triza-7-phosphaadamantane).[20]

Pathway b in Scheme I also explains the reactivity with disulfide if the anionic gold complex is activated towards disulfide exchange. It is known that increasing the electron density on a transition metal complex activates it toward oxidative addition.[21] In addition, many transition metals undergo oxidative addition reactions with organic disulfides. A plausible mechanism that accounts for both the conductivity of dppm$(AuSC_6H_4CH_3)_2$ and reactivity with disulfide is shown in Scheme II (where R = $C_6H_4CH_3$ and R* = $C_6H_4Cl$). The gold-gold interaction influences the acid-base properties of the dppm ligand and we propose that an anionic intermediate forms which is activated toward reaction with disulfide. Scheme II shows "oxidative addition" of disulfide occurring at one gold center, followed by "reductive elimination" of mixed disulfide and concomitant formation of the mixed gold complex. Small molecules, such as $CH_3I$ and $I_2$, are known to oxidatively add across two gold(I) atoms in dinuclear complexes.[22] However, if disulfide oxidatively adds across the golds in this case, it would lead to an equal probability of formation of symmetrical and mixed disulfides. Oxidative addition to one gold center is more consistent with the experimental results because the mixed disulfide forms significantly earlier in the reaction than does the symmetrical disulfide, $(p\text{-}SC_6H_4CH_3)_2$. Finally, it is interesting and somewhat unusual that the oxidative addition product is unstable and apparently immediately undergoes reductive elimination of disulfide.[22,23] We assume that the reaction is governed by the formation of the most thermodynamically stable disulfide and gold-thiolate bonds. We are continuing to investigate the role of auriophilic Au-Au interactions in the deprotonation of the dppm ligand as well as in the reaction with disulfides.

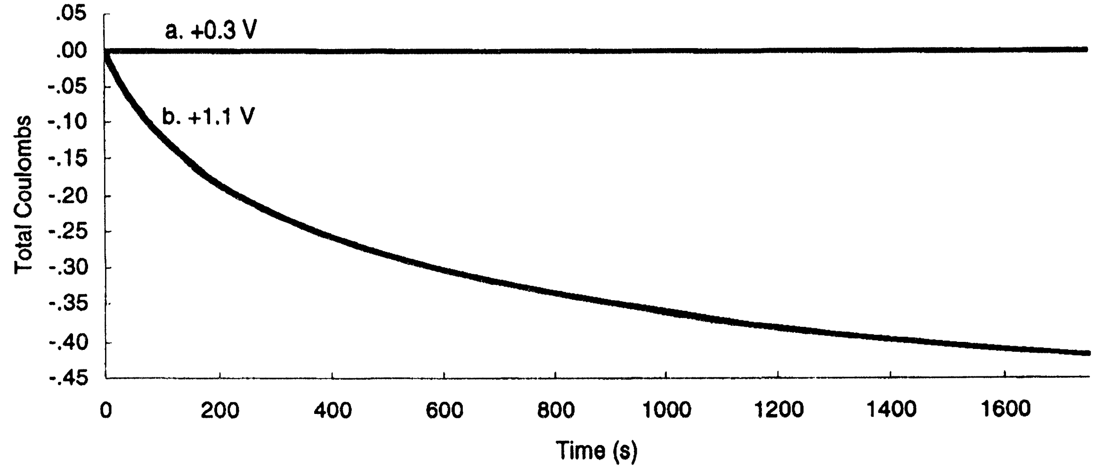

**Figure 2.** Bulk Electrolysis of $dppm(AuSC_6H_4CH_3)_2$ in 0.1 *M* $TBAH/CH_2Cl_2$. (a) +0.3 V, (b) +1.1 V (vs. SCE).

**Scheme II**

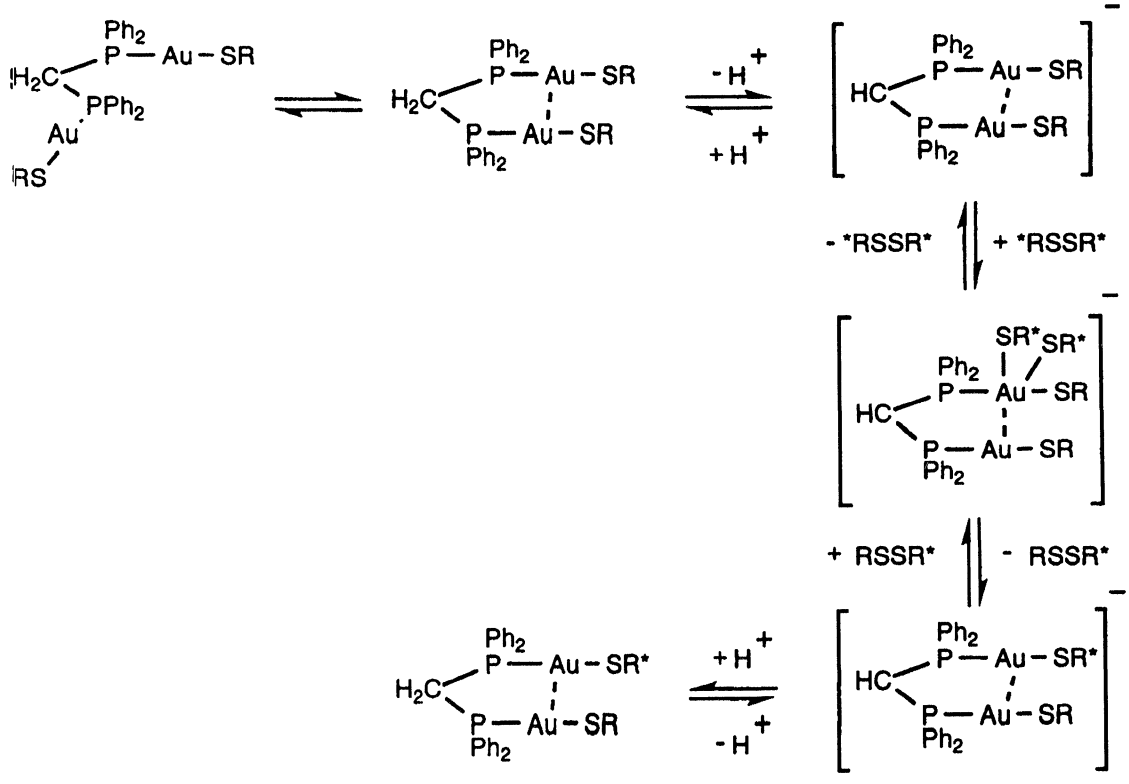

**ACKNOWLEDGEMENT**

Alfa Aesar (Johnson Matthey) is acknowledged for a generous loan of $HAuCl_4$.

### REFERENCES

1. Puddephatt, R. J. *The Chemistry of Gold*, Elsevier, NY, (1978).
2. (a) Hill, D. T.; Lantos, I.; Sutton, B. M. U.S. Patent 4,114,642, Sept. 19, 1978. (b) Reglinksi, J.; Smith, W. E. *Inorg. Chim. Acta*, **152**, 261-264 (1988). (c) Wang, S.; Fackler, J. P., Jr. *Inorg. Chem.* **29**, 4404-4407 (1990). (d) Hofreiter, S.; Paul, M.; Schmidbaur, H. *Chem. Ber.*, **128**, 901-905 (1995). (e) Roberts, J. R.; Xiao, J.; Schliesman, B.; Parsons, D. J.; Shaw, C. F., III *Inorg. Chem.* **35**, 424-433 (1996). (f) Xiao, J.; Shaw, C. F., III *Inorg. Chem.*, **31**, 3706-3710 (1992).
3. For several leading references concerning metal thiolate-disulfide interchange see: (a) Dean, P. A. W.; Jagadese, J. *Inorg. Chem.*, **25**, 514-519 (1986) (b) Zhu, Z.; Petering, D. H.; Shaw, C. F, III *Inorg. Chem.* **34**, 4477-4483 (1995).
4. Foley, J.; Fort, R.C., Jr.; McDougal, K.; Bruce, M.R.M.; Bruce, A.E. *Metal-Based Drugs*, **1**, 405-417 (1994).
5. Ganesh, S. "Reactions of Gold(I) Phosphine Thiolate Complexes with Organic Disulfides" University of Maine, M.S. Thesis, 1995.
6. (a) Fava, A.; Reichenbach, G.; Peron, U. *J. Am. Chem. Soc.*, **89**, 6696-6700 (1967). (b) Oswald, A. A.; Wallace, T. J. Organic Sulfur Compounds; Vol. II, Pergamon Press, Ltd.: London, 1966. (c) Kosower, E. M. in *Glutathione: Chemical, Biochemical and Medical Aspects*, Part A; Dolphin, D.; Arramovic, O.; Eds.; Wiley-Interscience: New York, 1989, Chapter 4. (d) Lees, W. J.; Whitesides, G. M. *J. Org. Chem.* **58**, 642-647 (1993) and references therein. (e) Sungh, R.; Whitesides, G. M. *J. Am. Chem. Soc.*, **112**, 6304-6309 (1990). (f) Houk, J.; Whitesides, G. M. *Tetrahedon*, **45**, 91 (1989). (g) Houk, J.; Whitesides, G. M. *J. Am. Chem. Soc.*, **109**, 6825-6836 (1987). (h) Kice, J. L.; Slebocka-Tilk, H. *J. Am. Chem. Soc.*, **104** 7123-7130 (1984).
7. (a) Oae, S. *Organic Chemistry of Sulfur*, Plenum Press, NY, (1997). (b) Mukaiyama, T.; Takahashi, K. *Tet. Lett.*, **56**, 5907-5908 (1968).
8. Narayanaswamy, R.; Young, M.A.; Parkhurst, E.; Ouellette, M.; Kerr, M.E.; Ho, D.; Elder, R.C.; Bruce, A.E.; Bruce, M.R.M. *Inorg. Chem.*, **32**, 2506-2517 (1993).
9. Dean, J. *Analytical Chemistry Handbook*, McGraw Hill, 133-135 (1995).
10. Dilorenzo, M. "Gold(I) Mediated Thiolate/Disulfide Exchange Reactions" University of Maine, M.S. Thesis, 1995.
11. DiLorenzo, M.; Ganesh, S.; Tadayon, L.; Chen, J.; Bruce, M. R. M.; Bruce, A. E. unpublished results.
12. Two replications were carried out using 4 different concentrations (2-16 mM) each of dppm$(AuSC_6H_4CH_3)_2$ and $ClC_6H_4SSC_6H_4Cl$, for a total of 16 experiments.
13. We assumed that at the lowest concentration measured for [Au(dppee)2][PF6] it behaves as an strong electrolyte. The ratio of the conductance of dppm$(AuSC_6H_4CH_3)_2$ to [Au(dppee)2][PF6] then gives an estimate of the extent of ionization of dppm$(AuSC_6H_4CH_3)_2$. Although [Au(dppee)2][PF6] deviates from strong electrolyte behavior at higher concentration, to a first approximation, [Au(dppee)2][PF6] can be used as a reference electrolyte in the concentration range under investigation.
14. Foley, J. B.; Gay, S. E.; Turmel, C.; Wei, G.; Jiang, T.; Narayanaswamy, R.; Foxman, B. M.; Vela, M. J.; Bruce, A. E.; Bruce, M. R. M. *Metal-Based Drugs*, submitted.
15. B. Andrieux, C. P.; Hapiot, P.; Pinson, J.; Saveant, J.-M. *J. Am. Chem. Soc.*, **115**, 7783-7788 (1993).
16. Wilker, J. J.; Lippard, S. J. *J. Am. Chem. Soc.*, **117**, 8682-8683 (1995).
17. (a) Hormann, A. L.; Shaw, C. F., III; Bennett, D. W.; Reiff, W. M. *Inorg. Chem.*, **25**, 3953-3957 (1986). (b) Hormann-Arendt, A. L.; Shaw, C. F., III *Inorg. Chem.*, **29**, 4683-4687 (1990).
18. For an example of deprotonation of dppm coordinated to gold(I) see Fernandez, E. J.; Gimeno, M. C.; Jones, P. G.; Laguna, A.; Laguna, M.; Lopez-de-Luzuriaga, J. M. *Organometallics*, **14**, 2918-2922 (1995).

19. (a) Harwell, D. E.; Mortimer, M. D.; Knobler, C. B.; Anet, F. A. L.; Hawthorne, M. F. *J. Am. Chem. Soc.*, **118**, 2678-2685 (1996). (b) Schmidbaur, H. *Gold Bull.*, **23**, 11-20 (1990).

20. Assefa, Z.; McBurnett, B. G.; Staples, R. J.; Fackler, J. P., Jr.; Assmann, B.; Angermaier, K.; Schmidbaur, H. *Inorg. Chem.*, **34**, 75-83 (1995).

21. Collman, J. P.; Hegedus, L. S.; Norton, J. R.; Finke, R. C. *Principles and Applications of Organotransition Metal Chemistry*, University Science Books,: Mill Valley, CA, 1987.

22. Fackler, J. P., Jr. *Polyhedron*, **16**, 1-17 (1997).

23. Dyadchenko, V. *Russ. Chem. Rev.*, **51**, 265-271 (1982).

**Received: October 3, 1998 - Accepted in final form: March 1, 1999**

